# Recurrent Arterial and Venous Thromboemboli as Initial Presentation of Acute Promyelocytic Leukemia

**DOI:** 10.14740/jocmr1864w

**Published:** 2014-07-28

**Authors:** Felix Trottier-Tellier, Madeleine Durand, Christophe Kolan, Robert Wistaff, Paul Van Nguyen, Mikhael Laskine

**Affiliations:** a Universite de Montreal, Montreal, Canada; b Department of Medicine, Hopital Hotel-Dieu, CRCHUM, Montreal, Canada; c Department of Medicine, Hopital Hotel-Dieu, CHUM, Montreal, Canada

**Keywords:** Acute promyelocytic leukemia, All trans-retinoic acid and arsenic treatment, Arterial and venous thrombosis, Systemic anticoagulation

## Abstract

We report a case of a 52-year-old Caucasian woman diagnosed with a synchronic arterial and venous thrombosis as an initial presentation of an acute promyelocytic leukemia (APL). After the diagnosis, the patient was treated with all trans-retinoic acid and arsenic chemotherapy concomitant to systemic anticoagulation. This treatment regimen led to a complete remission and absence of relapse of the thrombosis or APL during the follow-up. To our knowledge, this presentation is the second case in the literature. We use this opportunity to emphasize the importance of performing a complete medical evaluation in cases of unusual thromboembolic events.

## Introduction

Vascular thromboemboli are among the most common medical problems presenting in the adult population. Large-scale retrospective studies show an incidence of first-time venous thromboemboli (combined deep venous thrombosis (DVT) and pulmonary embolism (PE)) of 1.92 per 1,000 persons per year with a recurrence rate of 7.7% per year [1]. Arterial events are even more common, with a prevalence for adult populations of 3-10% for peripheral artery disease and 2.8% for stroke [2, 3]. Although often considered distinct pathological entities, arterial and venous thrombosis have much in common. They share risk factors such as age, obesity, diabetes and hereditary or acquired thrombophilias [4, 5]. Current studies also suggest that a history of venous thromboembolic disease represents a significant risk of future cardiovascular disease, suggesting that arterial and venous thromboembolisms share common pathways [6, 7]. Nonetheless, very few diseases present with both arterial and venous thromboembolisms. Among these, classical causes include antiphospholipid syndrome, heparin-induced thrombocytopenia (HIT), Bechet’s syndrome, neoplasia-associated thrombosis, and myeloproliferative disorders. In this case report, we describe an unusual combined arterial and venous thrombosis and we emphasize on the medical evaluation required by such a case.

## Case Report

A 52-year-old female presented to the emergency department for acute pain and numbness in the left arm. She was known for arterial hypertension and gout, and was a former 30 pack-year smoker and obese (BMI 37). Two weeks prior to this presentation, she had been diagnosed with extensive DVT of unknown origin of the right leg (popliteal, tibial posterior and great saphenous veins) with bilateral legs superficial venous thrombosis. She was started on enoxaparin 1 mg/kg SC BID.

On arrival, she complained of intense left hand pain that suddenly developed a few hours earlier, accompanied by progressive left hand weakness. She denied having night sweats, fever, recurrent infections or bleeding. She had noted a slight subjective weight loss and fatigue in the last 6 weeks. No neurological, mucocutaneous or visual symptoms were reported. She was compliant with her medication.

She was hemodynamically stable. A complete physical examination was unremarkable, except for the exquisite left hand pain with coldness, pallor and pulselessness up to the elbow.

Acute left hand ischemia was suspected and an angiography scanner was performed. It confirmed the diagnosis of acute thrombosis of the left axillary artery and chronic thrombosis of the left subclavian artery with occlusive emboli in distal left ulnar artery. She underwent local arterial thrombolysis and then was started on IV heparin with fast symptoms improvement.

**Figure 1:**
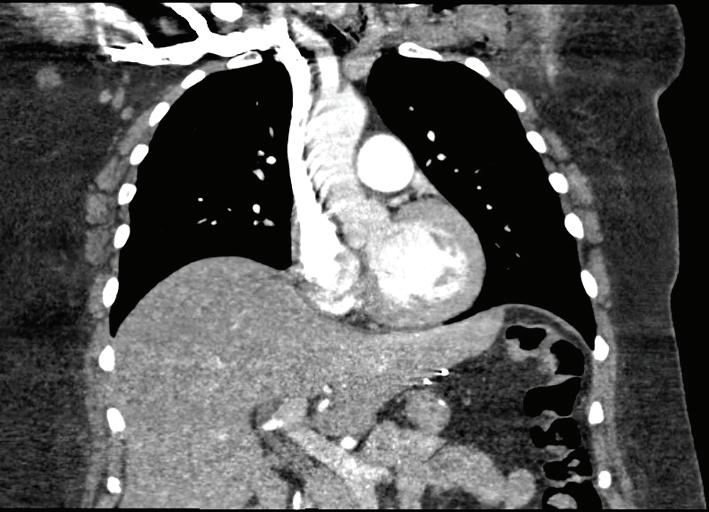
CAT scan showing thrombosis of the left subclavian and axillary arteries.

Local thrombolysis was performed twice again over the next 10 days because of recurrent episodes of left arm ischemia with thrombi progression in the brachial, radial and ulnar arteries. The decision to switch to IV argatroban was made due to repeated episodes of thrombosis with therapeutic levels of IV heparin, thus suspecting a “heparin resistance” (Fig. 1).

Unfortunately, the left upper limb ischemia persisted after the third local arterial thrombolysis and the patient undergone a surgical embolectomy of left radial and ulnar arteries with left carotido-humeral bypass. Signs and symptoms of ischemia rapidly recovered post-operatively and IV argatroban was switched to subcutaneous fondaparinux.

Parallel to the treatment of these recurrent episodes of large-sized vessels arterial and venous thromboemboli, we were looking for the possible etiology.

The blood tests showed a severe normocytic anemia with 75 g/L of hemoglobin, MCV 94 fL and severe neutropenia with 300 × 10^6^ neutrophils/L. There were no circulating blast cells.

We excluded an HIT syndrome (no thrombocytopenia and no anti-PF4-heparin antibodies) and an antiphospholipid syndrome (no beta-2-glycoprotein, no anticardiolipin antibodies and no lupus anticoagulant). There was no hyperhomocysteinemia with a homocysteine level of 4.5 µmol/L (normal 4 - 10). A hyperviscosity disorder was unlikely with no monoclonal gammopathy on protein electrophoresis and a normal kappa-lambda ratio. Disseminated intravascular coagulation (DIC) was excluded given an above upper limit fibrinogen value of 5.26 g/L (normal 2 - 4) and an absence of thrombocytopenia and schistocytes on blood smear.

A left saphenous vein biopsy and a left brachial artery thrombi biopsy did not show any signs of vasculitis. Moreover, a full body PET scan did not show any suspicious hypermetabolism area and a thoraco-abdominal scan was normal except for the previously stated thrombosis. Therefore, these imaging studies and biopsies allowed us to exclude large vessels vasculitis and solid tumor.

**Figure 2:**
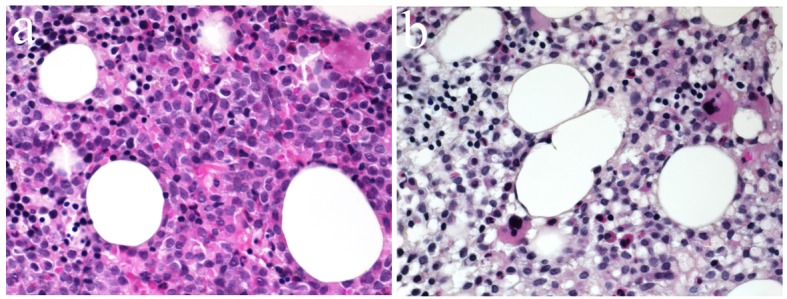
(a, b) Bone marrow biopsy result compatible with AML showing a hypercellular marrow with excess of immature myeloid cells and the presence of rare megacaryocytes.

Given the unexplained severe anemia and neutropenia, a bone marrow biopsy was performed. Preliminary result showed 40% blast cells, absence of AUER rod but strongly positive peroxidase stain, suggestive of acute myeloid leukemia of undefined classification. Molecular analysis confirmed the t(15:17) genetic anomaly and the diagnosis of low risk acute promyelocytic leukemia (APL) with secondary multiple arterial and venous thrombosis was done (Fig. 2).

**Figure 3:**
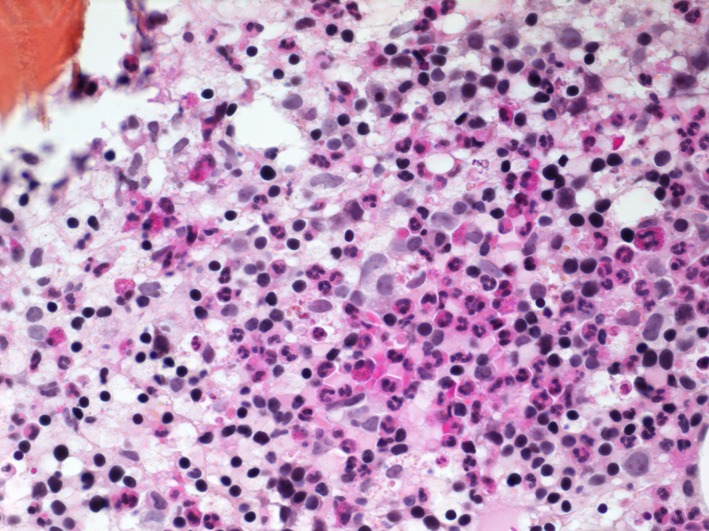
A normocellular bone marrow is observed after 1 month of treatment.

An induction treatment of all trans-retinoic acid (ATRA) and arsenic was immediately started. The patient had an excellent clinical evolution without evidence of the retinoic acid syndrome. As planned in our hospital protocol, a control bone marrow biopsy was performed after 1 month of induction treatment and confirmed a hematologic complete remission (Fig. 3).

The patient was sent home with a consolidation treatment of arsenic and ATRA. No signs of recurrence were observed after two cycles of consolidation. At the time of discharge, the cause of the recurrent thrombosis was clearly identified as APL. This pathology is known for its strong thrombogenic potential and it led to multiple recurrent thrombosis while on therapeutic dose of IV heparin with suspicion of “heparin resistance”. Once APL was confirmed to be in complete remission, the cause of these severe thrombosis was therefore controlled and the patient was switched back to LMWH enoxaparin 1.5mg/kg DIE SC for long-term anticoagulation. There was no recurrence of thromboembolic events thereafter.

## Discussion

APL is a hematopoietic neoplasia of the acute myeloid leukemia (AML) group. This disorder is characterised by an excess of atypical promyelocytes in the bone marrow and peripheral blood. The cytogenetic anomaly t(15:17) is a classic finding and is used for diagnostic confirmation of APL according to the WHO classification system of AML [8]. This translocation induces a fusion between promyelocytic leukemia (PML) gene and retinoic acid receptor alpha (RARalpha) gene creating a PML-RARalpha gene inducing excessive medullary production of promyelocytes through a PML-RARalpha protein. Five to twenty percent of AML cases are classified as APL [9]. The natural evolution of APL is very aggressive with median survival of less than 1 month, but recent advances in therapy with combined treatment of ATRA and arsenic provide excellent response with complete remission up to 90-95% of patients [10]. By binding to RARalpha moiety of the PML-RARA oncoprotein, ATRA leads to differentiation from promyelocytic malignant cells to mature neutrophils while arsenic binds to the PML moiety and induces apoptosis and partial differentiation of the promyelocytes cells [11, 12].

APL has been associated with important coagulopathy, which can present by bleeding or thrombosis. Hemorrhagic complications have been recognized for decades as an important clinical feature of APL and as the leading cause of early mortality [13]. The pathogenesis of APL coagulopathy is incompletely understood. Initially attributed to DIC, recent publications have shown a complex interaction of coagulation activation and hyperfibrinolysis in APL considerably different from DIC [14]. Thrombosis complications, although increasingly identified as part of the APL coagulopathy spectrum, are less understood and underreported compared with bleedings. Nevertheless, elevated white blood cell count, presence of FLT3-ITD and expression of CD2 were found to be associated with an increased risk of thrombosis [15]. Pathogenesis hypotheses of APL-related thrombosis are direct expression of tissue factor and cancer procoagulant and prothrombogenic cytokines production by abnormal promyelocytes. Concerns have been raised since it was observed that APL thrombosis events are more frequently reported since the introduction of ATRA. Although not fully understood, direct effects of ATRA that might promote thrombosis include increased production of endothelial thrombogenic stimulating cytokines by the APL cells and increased adhesion of the APL cells to endothelial cells [16].

Both venous and arterial thromboembolic events have been described with APL. A recent review article reported 94 cases of major APL-related thrombosis [17]. The majority (84%) of these thrombosis occurred before or during induction therapy and 55% were arterial compared with 45% venous. The most frequent locations of thrombosis were DVT/PE (28.7%), cardiac events (26.6%) and strokes (21.3%) for a combined total of 76.6% of the thrombotic events. Of note, five patients presented acute limb ischemia and six patients presented with multiple thrombotic events including two combined venous and arterial thrombosis (one splenic infarct and DVT and one acute limb ischemia and PE). In only one of these two cases, thrombosis was the initial complain.

Therefore, the case presented in this paper was a rare presentation of APL. To our knowledge, it is only the second case reported of acute arterial thrombosis combined with DVT/EP as a presentation of APL. Unfortunately, the diagnosis was delayed by the absence of blast cells in peripheral blood on initial examination. Nevertheless, treatment was rapidly successful for the APL per se and for the thromboembolic events once the combined ATRA and arsenic induction protocol was started.

As stated above, obesity has been recognized as an important risk factor for both venous and arterial thrombosis. The patient presented here had class II obesity, which could certainly contribute to the clinical thrombosis. As the medical investigation demonstrated, the obesity was not the primary cause of the thrombotic events and it would be highly unlikely that long standing obesity would be the main cause of brutal recurrent thrombosis.

In conclusion, venous and arterial thromboembolic events are among the most common medical challenges encountered by internal medicine teams. A complete investigation such as the one described in this case report would be a resource misuse if applied to a majority of thromboembolic cases. However, a search for the cause of a thrombosis should always be undertaken and must be tailored to each patient’s presentation. This investigation could go from anamnesis only to extensive laboratory and imaging procedures. We believe it is our role as internists to offer a thorough medical evaluation when challenged with recurrent or unusual presentation of thromboembolic arterial or venous events. In this context, some of the most important etiologies to investigate would include solid and hematologic neoplasia, vasculitis, thrombophilias, HIT syndrome and antiphospholipid syndrome. An accurate diagnosis is of prime value since these various pathologies present widely different prognosis and often require specific management besides anticoagulation.

We feel this case report is a perfect example to stress the importance of a rigorous and complete investigation that some thromboembolic events call for. The goal of this paper is to help medical teams keep a high level of suspicion when facing unusual thromboembolic events and improve the knowledge concerning APL-related coagulopathy and its increased risk of thrombosis.
